# Parishin A ameliorates cognitive decline by promoting PS1 autophagy in Alzheimer’s disease

**DOI:** 10.3389/fnagi.2025.1516190

**Published:** 2025-03-20

**Authors:** Song Guo, Lilin Yi, Man Luo, Zhifang Dong, Yehong Du

**Affiliations:** Pediatric Research Institute, Ministry of Education Key Laboratory of Child Development and Disorders, National Clinical Research Center for Child Health and Disorders, Chongqing Key Laboratory of Child Neurodevelopment and Cognitive Disorders, Children’s Hospital of Chongqing Medical University, Chongqing, China

**Keywords:** Alzheimer’s disease, Parishin A, amyloid-β, PS1, autophagy

## Abstract

**Introduction:**

Alzheimer’s disease (AD) is a common neurodegenerative disease in the elderly. Its pathological features include: A lot of misfolding and abnormal aggregation of amyloid protein (Aβ); Autophagy disorder, oxidative stress, neuroinflammation, abnormal phosphorylated tau protein and synaptic dysfunction. Modern pharmacological studies have found that Paisinhin A (PA) has beneficial effects on the prevention and treatment of central nervous system diseases. This study aims to explore the role and mechanism of PA in AD through autophagy pathway, and lay a scientific foundation for the development of clinical prevention and treatment strategies for AD.

**Methods:**

N2A^APP^ cells were treated with different concentrations of PA. Cell viability was detected by CCK-8 method. Western blotting detected the expression levels of proteins related to amyloid production, autophagy pathway, and phosphorylated Tau expression levels. Autophagy flow was detected by transfecting Lc3 double fluorescent plasmid. After Aβ was injected into the hippocampus of WT mice and PA was injected intraperitoneally, the learning and memory ability of WT mice were tested by new object recognition, y maze and water maze. The oxidative stress level was detected by the kit. The levels of inflammatory factors were detected by RT-qpcr.

**Results:**

The viability of N2A^APP^ cells was not affected at different concentrations of PA, but PS1 was significantly decreased at 40μM. PA can obviously improve the accumulation of autophagy in AD, and to some extent save the autophagy inhibition of CQ. Behavioral studies have shown that PA can also improve learning and memory impairments caused by Aβ injections. In addition, *in vivo* experiments, PA can also improve oxidative stress levels, inflammation levels and salvage dysfunctions of synapses. PA also reduces the levels of total and phosphorylated Tau in N2A^Tau^.

**Discussion:**

Our study provides the first evidence that PA improves learning and memory in Aβ-induced AD mice. This effect appears to be mediated by PA by promoting autophagy and reducing oxidative stress. It was also found that PA may have a role in regulating inflammation, improving abnormally phosphorylated tau, and salvaging damaged synaptic function, providing valuable insights into potential applications in the treatment and prevention of AD.

## Graphical abstract

Parishin A enhances autophagy, leading to reduced PS1 expression and thereby improving learning and memory in AD mice.
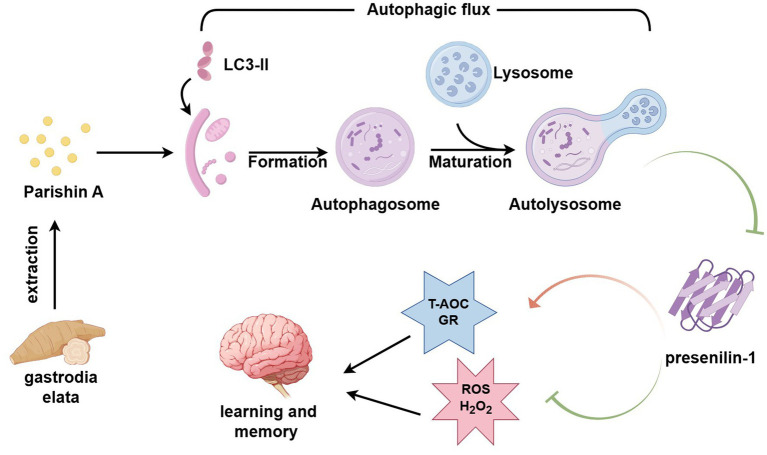

## Introduction

Alzheimer’s disease (AD) is the most common cause of dementia, currently affecting more than 55 million people worldwide, with this number projected to rise to 139 million by 2050 (World Alzheimer Report, 2023). The accumulation of amyloid beta (Aβ) plaques, produced by the sequential cleavage of amyloid precursor protein (APP) by β-secretase (BACE1) and γ-secretase, is a neuropathological hallmarks of AD (Graff-Radford et al., 2021). Presenilin-1 (PS1), a catalytic component of the γ-secretase complex, is responsible for cleaving APP to generate Aβ peptides of varying lengths, including Aβ40 and the more pathogenic Aβ42. The ratio of these peptides is influenced by PS1 activity (Bagaria et al., 2022).

The degradation of Presenilin 1 (PS1) is crucially mediated by several pathways, including the Ubiquitin-Proteasome Pathway (UPP) and the autophagy-lysosome pathway, which are vital for maintaining its function and preventing abnormal accumulation of Aβ (Pasternak et al., 2003). PS1 undergoes ubiquitination by SEL-10 and tumor necrosis factor receptor-associated factor 6 (TRAF6) (De Strooper and Annaert, 2010). Furthermore, PS1 features a ubiquitin-binding CUE domain that specifically enhances its interaction with K63-linked polyubiquitin chains, thus facilitating its regulation via these degradation pathways. Proteins ubiquitinated with K63 chains, such as PS1, can act as substrates for autophagy receptors like p62/SQSTM1. These receptors detect K63-linked ubiquitin and escort the associated proteins to the autophagosome. Conversely, autophagy impairment stimulates PS1 expression, indicating that autophagy-lysosome pathway is a crucial way for PS1 degradation.

In addition to Aβ deposition, hyperphosphorylation of Tau also plays a crucial role in AD pathogenesis. Some evidence indicates that Aβ accumulation alone does not consistently correlate with cognitive decline, as many older adults with brain Aβ deposits do not progress to AD (Lozupone et al., 2024; Yoon et al., 2024). Tau abnormalities, which can be triggered by Aβ, may also independently cause neurological damage, and Tau pathology can persist even after Aβ clearance (Gao et al., 2018). Research advances have increasingly acknowledged Tau proteins as clinical biomarkers for AD. Plasma p-Tau181, p-Tau217, and p-Tau231 are recognized as early diagnostic markers, showing high consistency with PET and CSF results (Leuzy et al., 2021). Additionally, a biomarker known as Alz-Tau^®^ has been developed to effectively differentiate AD patients from healthy individuals by analyzing the ratio of high molecular weight Tau (HMWTau) to low molecular weight Tau (LMWTau) in platelets (Marizzoni et al., 2023). This Tau-focused monitoring aids early diagnosis, serves as an indicator of disease progression and therapeutic response, and supports AD management and new therapy development.

APOE is a gene that encodes a protein in the brain involved in lipid metabolism. There are three common APOE subtypes: epsilon 2, epsilon 3 and epsilon 4. The epsilon 4 subtype has been linked to an increased risk of developing AD (Cao et al., 2024). APOE4 carrier status is associated with increased risk of AD through amyloid - and tau-dependent and independent pathways, such as increased neuroinflammation (Burt et al., 2008; Wang et al., 2022), disruption of glucose and lipid metabolism, causing oxidative stress, and reduced neurovascular integrity (Yamazaki et al., 2019). In addition, the APOE genotype may interact with diet to further influence the risk of AD (Yassine and Finch, 2020). At the same time, APOE may be independently associated with lifestyle spectrum and cognitive impairment in AD patients (Jin et al., 2021; Solomon et al., 2018). These results suggest that the entanglement of APOE with viral infection, environmental impact, etc. is interesting and worthy of further investigation.

Parishin, derived from Chinese herbs, exhibits a range of pharmacological properties including anti-inflammatory, antioxidant, and antipsychotic effects (Lin et al., 2016). Parishin A (PA), a phenolic glucoside isolated from Gastrodia elata, is a key component in traditional Chinese medicine. Studies have shown that PA significantly reduces aging-related markers and inhibits the expression of inflammatory factors while promoting M2 polarization of macrophages. Although there is no clear evidence relating PA’s effects to autophagy, it has been observed that in tendon stem/progenitor cells, PA can inhibit the excessive activation caused by rapamycin, which regulates the secretion of inflammatory cytokines by macrophages (Zhu et al., 2023). However, the mechanisms underlying PA’s potential therapeutic benefits in AD are not well understood. In this study, we explore the neuroprotective effects of PA in countering the decline of autophagy in AD.

## Materials and methods

### Animals

C57BL/6 male mice (25–30 g) (Beijing Vital River Laboratory Animal Technology Co., Ltd) were housed in an animal care facility at the Children’s Hospital of Chongqing Medical University under standard laboratory conditions, which included temperature and humidity control, individual housing in plastic cages, and a 12-h light/dark cycle. The mice were given ad libitum access to food and water. All animal experiments were conducted by the Chongqing Science and Technology Commission guidelines and approved by the Chongqing Medical University Animal Care Committee. Every effort was made to minimize animal suffering and the number of animals used.

### Drugs preparation and administration

Aβ_1–42_ peptides (A9810, Sigma, USA) were initially dissolved in hexafluoroisopropanol (HFIP) (AG968, Sigma, USA) and left at room temperature for 60 min. The resulting peptide film was then dissolved in dimethyl sulfoxide (DMSO) (D2650, Sigma, USA) to achieve a concentration of 5 mM. The solution was diluted to 100 μM with phosphate-buffered saline (PBS) and incubated at 4°C for 48 h to form oligomers.

The lateral ventricular administration of Aβ_1–42_ is a commonly used experimental method to replicate the pathophysiology of Alzheimer’s disease (AD) (Du et al., 2019). Following anesthesia with 60 mg/kg sodium pentobarbital (i.p.), the mice’s cranial hair was removed, and their heads were secured in a stereotactic device in a horizontal position. The scalp was disinfected with povidone-iodine solution, and a midline incision was made to expose the skull. 2.5 μL of Aβ_1–42_ (100 μM) was administered at a rate of 0.5 μL/min, targeting coordinates −0.5 mm posterior, +1.1 mm lateral, and − 3.0 mm ventral relative to bregma. The syringe was left in place for 5 min to ensure complete diffusion of the fluid. After carefully removing the syringe and suturing the scalp, the mice were returned to their cages for rewarming.

Parishin A was (HP265836, Chenguang Biology, Baoji, Shanxi, China dissolved in sterile PBS). Animals were randomly assigned to different treatment groups: WT, WT + Aβ, and WT + Aβ + PA. The WT + Aβ + PA group received an intraperitoneal injection of PA (10 mg/kg) starting 1 week before the Aβ_1–42_ microinjection and continuing until the completion of the behavioral tests. The WT and WT + Aβ groups were administered the same volume of PBS.

Mice received intraperitoneal injections of PA starting at 8 weeks of age, followed by lateral ventricular administration of Aβ_1–42_ at 9 weeks. Behavioral assessments were conducted at 11 weeks, and sacrifice with sample collection occurred at 12 weeks and 2 days.

### Novel object recognition

Three weeks after the intraperitoneal injection of PA, mice underwent the Novel Object Recognition (NOR) test. During the training phase, two identical, odorless, and fixed objects (A and B) were placed in the apparatus, approximately 10 cm away from each side wall. Each mouse was introduced into the apparatus at an equal distance from the objects, with its back facing them. Exploration behavior, defined as touching the object with the mouth or nose or approaching within 2–3 cm, was recorded using video equipment and software. The number of interactions, exploration time, and distance traveled around each object were measured over a 5-min period. Following a 2-h interval, the test phase commenced, in which one of the identical objects was replaced with a novel object. The mouse was reintroduced into the apparatus under the same conditions, and exploration behavior was recorded for 5 min. All experiments were tracked and analyzed using the ANY-maze tracking system (Stoelting, USA).

### Y-maze

The Y-maze apparatus consisted of three opaque plastic arms, labeled A, B, and C, arranged at 120° angles to each other. Mice were placed at the center of the maze and allowed to explore for 5 min. Entry into an arm was counted when a mouse moved all four limbs into the arm. The sequence of arm entries, such as A-B-C or B-C-A, was recorded to calculate the alternation score. Between tests, the maze was cleaned with 75% alcohol, which was allowed to evaporate completely to eliminate odor interference. The experiments were documented using the ANY-maze tracking system from Stoelting, USA. This alternation score was determined using the formula: spontaneous alternation rate (%) = [(number of spontaneous alternations) / (total number of arm entries–2)] × 100.

### Morris water maze test

Three weeks after the intraperitoneal injection of PA, mice were subjected to the Morris Water Maze task. The test was conducted in a circular pool with a diameter of 150 cm and a height of 50 cm, filled with water maintained at 21–22°C, made opaque with nontoxic white paint. Light blue curtains surrounded the pool to create an isolated environment, with three geometric shapes attached to the curtains as visual cues. A high-definition camera was positioned directly above the pool to record the animals’ movements. A platform with a diameter of 10 cm was placed in the middle of the third quadrant of the pool, with the water level kept 1 cm above the platform. The day before spatial training, the mice were allowed to swim freely in a pool without a platform for 120 s to acclimatize. Subsequently, the mice underwent acquisition training for five consecutive days, with four trials per day. In each trial, mice that failed to locate the platform within 120 s were gently guided to it and allowed to stay there for 20 s. On the day following the final training session, the platform was removed, and the mice underwent a 120-s probe test. All experiments were recorded using the ANY-maze tracking system (Stoelting, USA).

### Mouse euthanasia and brain tissue harvesting

1.5% sodium pentobarbital 100 mL: Sodium pentobarbital 1.500 g was weighed precisely and placed into a beaker. Distilled water was then added to completely dissolve it and transferred into a 100 mL volumetric flask for constant volume. Sodium pentobarbital was prepared in a 1.5% solution of sterile saline at the usual dose of 30 mg/kg body weight, which translates to 0.2 mL per mouse. The administration of the drug was done gradually, especially more slowly after administering 3/4 of the planned dose, rather than all at once. During injection, the corneal reflex, muscle relaxation, and pain responses in the animals were closely monitored. Once adequate anesthesia was achieved, the drug injection was halted immediately.

Following the completion of behavioral experiments, the mice were euthanized using the previously described methods, and brain tissue was harvested as follows: The neck was severed with tissue scissors just behind the skull. The scalp was pulled aside, and a midline incision was made with a razor blade between the eyes. Thin scissors were inserted into the foramen magnum and used to cut laterally along the skull. Small incisions were then made from the midline toward the sides. Using forceps, the skull flaps were flipped toward the sagittal suture, and the procedure was repeated on the opposite side. The brain was bisected with a clean surgical blade.

To remove the hippocampus, two short microscopic tweezers were used. One tweezer was placed near the junction of the cerebellum and cortex, and the other was positioned at the same point to gently dissect the cortical hemisphere laterally, exposing the hippocampus. The brain was stabilized with one tweezer, while the other tweezer was moved slightly forward and laterally, applying careful pressure to the medial white matter tract of the hippocampus. The hippocampus was then scooped or rolled sideways onto filter paper with the smooth end facing up, and the procedure was repeated for the second hemisphere.

### Cell culture and treatment

Mouse neuroblastoma cells stably expressing human Swedish APP 695 (N2A^APP^), obtained from Professor Chunjiu Zhong (Fudan University, Shanghai, China) were cultured in a medium consisting of 90% Dulbecco’s modified Eagle’s medium (DMEM) (11960044, Gibco, USA), 10% fetal bovine serum (FBS) (10099, Gibco, USA), and 100 μg/mL of G418 (11811031, Gibco, USA) at 37°C in a 5% CO_2_ atmosphere.

To detect the effects of PA on the PS1 degradation, N2A^APP^ cells were pretreated with 40 μM PA or a control solvent for 12 h. This was followed by the treatment of 100 μg/mL cycloheximide (CHX) (CST, Danvers, MA, United States) to inhibit protein synthesis for various time (0, 6, 12 and 24 h) (Du et al., 2019). To investigate whether PA affects the ubiquitin-proteasome or autophagy lysosome degradation pathways of PS1, N2A^APP^ cells were pretreated with either 50 μM CQ (HY-17589A, MCE, USA) or MG132 (HY13259, MCE, USA) for 1 h, followed by treatment with 40 μM Parishin A for 24 h.

### Stable cell lines constructed

Cells were seeded in 10-well plates at a density that allowed them to reach about 70% confluency by the following day. The human Tau plasmid was transfected into N2A cells. Forty-eight hours post-transfection, puromycin was added to the medium to select for successfully transfected cells by eliminating the non-transfected cells. Approximately 2 weeks later, cells were reseeded into 96-well plates at a density of one cell per well. The cells were then allowed to grow until they reached the desired confluency.

### Cell viability assay

N2A^APP^ cells were seeded into 96-well plates and treated with gradient concentrations of PA (0, 20, 40, 80, 160, and 320 μM) for 24 h. Subsequently, 10 μL of Cell Counting Kit-8 (CCK-8) reagent (MCE, Shanghai, China) was added to each well and were incubated at 37°C for 3 h, protected from light. Cell viability was determined by measuring the absorbance at 450 nm using a microplate reader (Thermo Fisher Scientific, MA, USA). Each group was subjected to four independent experiments, with each experiment performed in triplicate.

### Antibodies

Anti-C20 (1:1000) antibody used to detect APP and its β-CTFs was kindly provided by laboratory of Professor Weihong Song. Anti-BACE1 (1:1000, #ab183612), anti-PS1 (1:1000, #ab76083) antibodies were purchased from Abcam (Cambridge, MA, USA). Anti-Ubquitin (1:1000, #10201-2-AP), β-actin (1:3000, 60004-1-1 g) antibodies were obtained from Proteintech (Wuhan, Hubei, China), Anti-P62 (1:1000, H00008878-M01), anti-LC3 (1:1000, #12741) antibodies were purchased from Abnova (Taipei, Taiwan, China), anti- p-tau(Ser199)(1:1000AF2418), anti- p-tau(Ser396) (1:1000, AF3148), anti- p-tau(Ser404)(1:1000, AF31440), anti- p-tau(Thr181) (1:1000, AF31449) antibodies were purchased from affinity (Affinity Biosciences, OH, USA) Anti-Tau5 (1:1000, A23490) antibodies were purchased from abclonal (Wuhan, Hubei, China), Anti-SYP (1:1000, MAB1598) was purchased from CST (Danvers, Massachusetts, USA).

### Western blot

The cells were washed with ice-cold phosphate-buffered saline (PBS) and lysed on ice for 30 min in RIPA lysis buffer (Beyotime, Shanghai, China) containing protease inhibitors (Roche, Basel, Switzerland). After centrifugation at 12,000 rpm for 15 min at 4°C, the supernatant was collected. Protein concentration was determined using a BCA Protein Assay Kit (Thermo Fisher Scientific, MA, USA). Subsequently, 30 μg of total protein was denatured by boiling with 5× sample buffer at 95°C for 5 min. The samples were then separated using Tris-glycine SDS-PAGE gels (EpiZyme, Shanghai, China) and transferred to Immobilon-PTM polyvinylidene difluoride (PVDF) membranes (Millipore, MA, USA). The PVDF membrane was blocked with 5% bovine serum albumin (Sigma, St. Louis, MO, USA) for 1.5 h to minimize nonspecific binding. The membranes were then incubated with primary antibodies overnight at 4°C, followed by incubation with corresponding HRP-labeled secondary antibodies (goat anti-rabbit IgG or goat anti-mouse IgG, both at 1:3000 dilution, Perkin-Elmer) for 1–2 h at room temperature. The blots were visualized using the Bio-Rad Imager with Western ECL substrate (Bio-Rad, Hercules, CA, USA). Immunoblotting with GAPDH was performed to ensure equal loading and protein quality. Band intensities were quantified using Bio-Rad Quantity One software (Bio-Rad, Hercules, CA, USA).

### Co-immunoprecipitation (Co-IP)

Cells were lysed using IP buffer (Beyotime, Shanghai, China) supplemented with protease inhibitors for 30 min, and the lysates were then centrifuged at 12,000 *g* for 15 min at 4°C to obtain the supernatants. A total of 500 μg of protein from each sample was incubated overnight at 4°C with an anti-PS1 primary antibody or nonspecific IgG as a control. This was followed by an incubation with protein A/G magnetic beads for IP (Millipore, MA, USA) for an additional 2 h at 4°C. After incubation, the beads were washed four times with ice-cold PBS. The proteins bound to the beads were eluted by boiling in 2× SDS-PAGE loading buffer at 95°C for 5 min. These eluted proteins were then analyzed by subsequent immunoblotting.

### Quantitative real-time PCR (qRT-PCR)

Total RNA from cells and brain tissues were extracted using High Pure Total RNA Extraction Kit (Bio Teke, Peking, China) in accordence with the manufacturer’s protocol. The concentration and purity of RNA were assessed utilizing a NanoDrop 2000 spectrophotometer (Nanodrop Technologies, Wilmington, DE, USA). Subsequently, 1 μg of total RNA served as a template to synthesize single-stranded complementary DNA (cDNA) using the PrimeScript™ RT Reagent Kit (Takara, Otsu, Shiga, Japan). The synthesized cDNA was amplified by quantitative real-time PCR (qRT-PCR) using SYBR Premix Ex Taq II (Takara, Otsu, Shiga, Japan) and managed through CFX Manager software (Bio-Rad, Hercules, CA, USA). The primer used were as follows: PS1 (forward: 5′-GAGACTGGAACACAACCATAGCC, reverse: 5′-AGAACACGAGCCCGAAGGTGAT); GAPDH (forward: 5′-GGCATTGTGGAAGGGCTCAT, reverse: 5′-AGATCCACGACGGACACATT). IL-4 (forward: 5′- GGTCTCAACCCCCAGCTAGT, reverse: 5′- GCCGATGATCTCTCTCAAGTGAT); IL-6 (forward: 5′- TAGTCCTTCCTACCCCAATTTCC, reverse: 5′- TTGGTCCTTAGCCACTCCTTC). GAPDH was employed as an internal control for normalization, and the relative mRNA levels of PS1 were normalized to those of GAPDH. To calculate the ΔCt value for each sample, subtract the Ct value of the housekeeping gene GAPDH from the Ct value of the target gene. Then, compute the ΔΔCt value by subtracting the average ΔCt of the reference sample from the ΔCt of each individual sample. Finally, the relative expression of each gene is calculated using the formula 2^−ΔΔCt^.

### Oxidative stress analysis

The brain tissues (cortex and hippocampus) from mice were homogenized to a 10% concentration in saline on ice, followed by centrifugation at 2500 rpm for 15 min at 4°C to collect the supernatant. The levels of pro-oxidants including hydrogen peroxide (H_2_O_2_), total nitric oxide synthase (T-NOS) and inducible nitric oxide synthase (i-NOS) as well as antioxidants including glutathione reductase (GR) and total antioxidant capacity (T-AOC) were measured using commercial assay kits (Jiancheng Biochemical, Nanjing, Jiangsu, China) according to the manufacturer’s instructions.

For the H_2_O_2_ assay, the supernatant was first mixed with reagents 1 and 2, incubated at 37°C for 1 min, followed by the addition of reagents 3 and 4. The mixtures were thoroughly mixed, and the absorbance at 405 nm was measured using a microplate reader.

For NOS detection, the supernatant was incubated with or without reagent 6 (for i-NOS and T-NOS, respectively), along with reagents 1, 2, and 3 at 37°C for 15 min. Reagents 4 and 5 were then added, mixed thoroughly, and the absorbance at 530 nm was recorded using a microplate reader.

For the GR assay, the supernatant was incubated with the working solution at 37°C for 30 s, and the initial absorbance (A1) was recorded at 340 nm. After a further incubation of 10 min at 37°C, a second absorbance (A2) was recorded. The level of GR was calculated as the difference between A2 and A1.

For T-AOC measurement, the supernatant was mixed with reagents 1, 2, and 3 and incubated at 37°C for 30 min. After adding reagents 4 and 5, the mixture was incubated for an additional 10 min at 37°C, and the final absorbance at 520 nm was recorded.

### Autophagy flux

The mCherry-GFP-LC3B plasmid was utilized to establish a dual fluorescence autophagy system for assessing autophagy flux. In brief, cultured cells in a 6-well plate were transfected with the mCherry-GFP-LC3B plasmid using Lipofectamine 3000 (L3000008, Invitrogen, USA) for 24 h. After transfection, the cells were pretreated with PA (40 μM) for 6 h, and either with or without CQ (50 μM) for 24 h (Le et al., 2023; Yang et al., 2024). Subsequently, the cells were fixed with 4% paraformaldehyde (PFA) (158127, Sigma, USA), and the cell sheet was sealed with an anti-fluorescence quench agent to preserve fluorescence. Cellular images were captured using a Nikon 90i fluorescence microscope. Autophagy was quantified by counting GFP-LC3 and mCherry-LC3 dots in more than 15 cells from three replicate experiments.

### Statistical analysis

The statistical analysis was performed using the SPSS software (Version 25.0), and the data were expressed as means ± SEM. Student’s t-test or one-way ANOVA was used to evaluate the results. The significance level was set at *p* < 0.05.

## Results

### PA inhibits the amyloidogenic processing of APP by attenuating PS1 expression in N2A^APP^ cells

**Figure 1:**
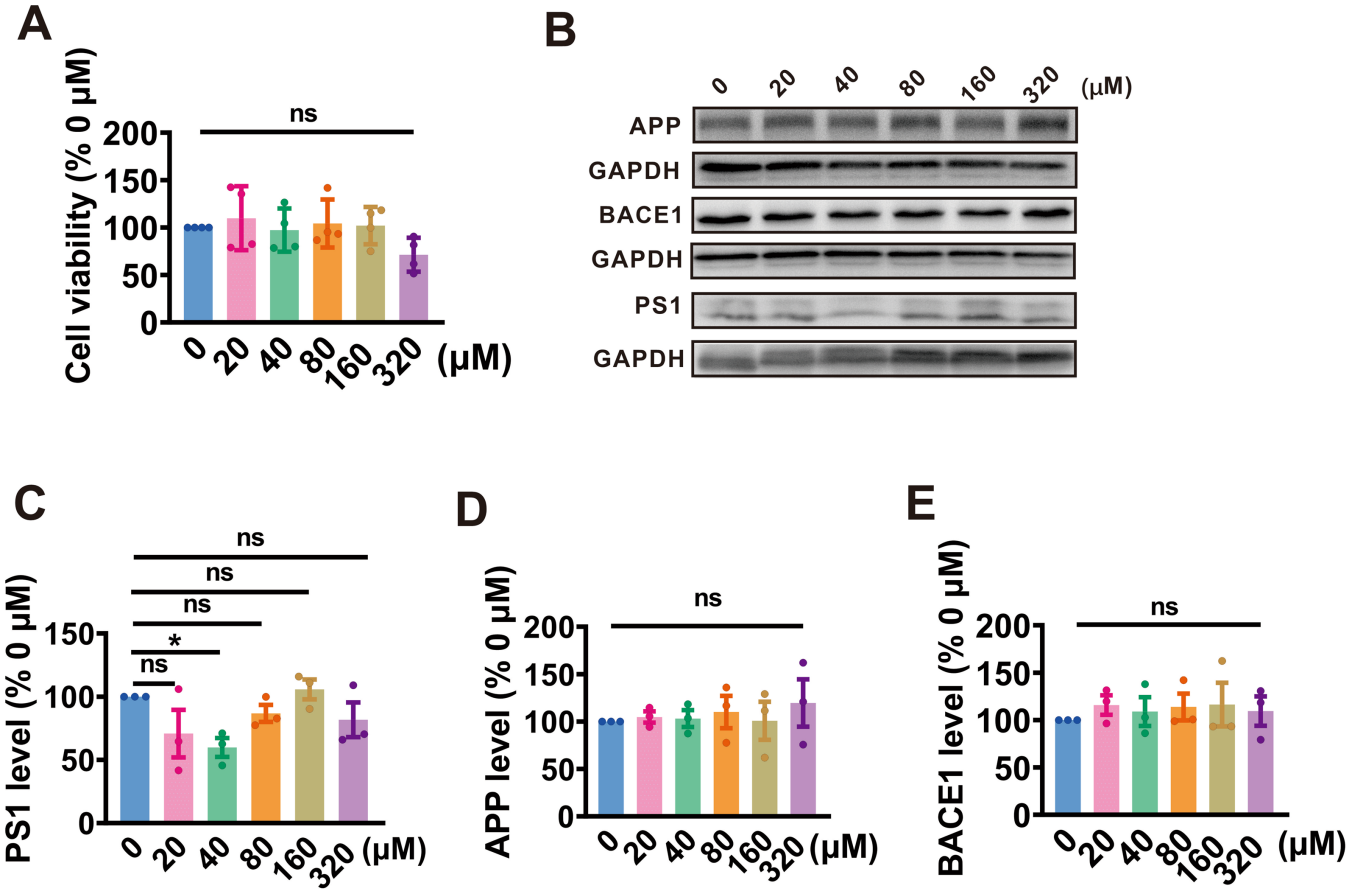
PA inhibits APP amyloidogenic processing by targeting PS1 in N2A^APP^ cells. **(A)** The cell viability assessed by CCK-8 in N2A^APP^ cells treated with a gradient concentration of PA (0–320 μM) for 24 h, *n* = 4 per group. **(B–E)** Protein levels of PS1 **(B,C)**, APP **(B,D)**, and BACE1 **(B,E)** assessed by Western blotting in N2A^APP^ cells were treated with gradient concentrations of PA (0–320 μM) for 24 h, *n* = 3 per group. Data are presented as mean ± standard error, *p < 0.05, **p < 0.01, ***p < 0.001.

To evaluate the impact of PA on cell survival, N2A^APP^ cells were exposed to gradient concentrations of PA (ranging from 0 to 320 μM) for 24 h. Cell viability was then assessed using the CCK-8 assay. It was observed that all concentrations of PA had no significant effect on the viability of N2A^APP^ cells, except for the highest concentration of 320 μM, which resulted in a decrease in cell viability (20 μM: 70.79% ± 18.0.82%, *p =* 0.801 vs. 0 μM; 40 μM: 59.78% ± 7.49%, *p =* 0.222 vs. 0 μM; 80 μM: 86.70% ± 6.74%, *p* = 0.403 vs. 0 μM; 160 μM: 105.70% ± 7.81%, *p* = 0.715vs. 0 μM; 320 μM: 81.76% ± 13.70%, *p* = 0.257 vs. 0 μM; *n* = 4 in each group; Figure 1A).

Further, we evaluated the effect of PA on APP processing in N2A^APP^ cells. The findings revealed that treatment with PA at a concentration of 40 μM led to a reduction in PS1 expression (20 μM: 70.79% ± 18.82%, *p* = 0.081 vs. 0 μM; 40 μM: 59.83% ± 7.49%, *p* = 0.022 vs. 0 μM; 80 μM: 86.70% ± 6.74%, *p* = 0.040 vs. 0 μM; 160 μM: 105.70% ± 7.80%, *p* = 0.715 vs. 0 μM; 320 μM:81.76% ± 13.7%, *p* = 0.257vs. 0 μM; *n* = 3 in each group; Figures 1B,C). However, PA had no obvious impact on APP expression across all tested concentrations (20 μM: 104.80% ± 5.93%, *p* = 830 vs. 0 μM; 40 μM: 103.10% ± 8.98%, *p* = 0.898 vs. 0 μM; 80 μM: 110.10% ± 17.11%, *p* = 0.653 vs. 0 μM; 160 μM: 100.90% ± 20.08%, *p* = 0.969 vs. 0 μM; 320 μM: 119.50% ± 24.96%, *p* = 0.289 vs. 0 μM; *n* = 3 in each group; Figures 1B,D) and BACE1 (20 μM: 115.90% ± 10.31%, *p* = 0.462 vs. 0 μM; 40 μM: 108.90% ± 15.21%, *p* = 0.676 vs. 0 μM; 80 μM: 113.90% ± 14.12%, *p* = 0.519 vs. 0 μM; 160 μM: 116.34% ± 23.04%, *p* = 0.450 vs. 0 μM; 320 μM: 109.54% ± 15.40%, *p* = 0.657 vs. 0 μM; *n* = 3 in each group; Figures 1B,E). These data suggest that PA alleviates APP processing by attenuating PS1 expression.

### PA promotes autophagy-lysosomal degradation of PS1

**Figure 2:**
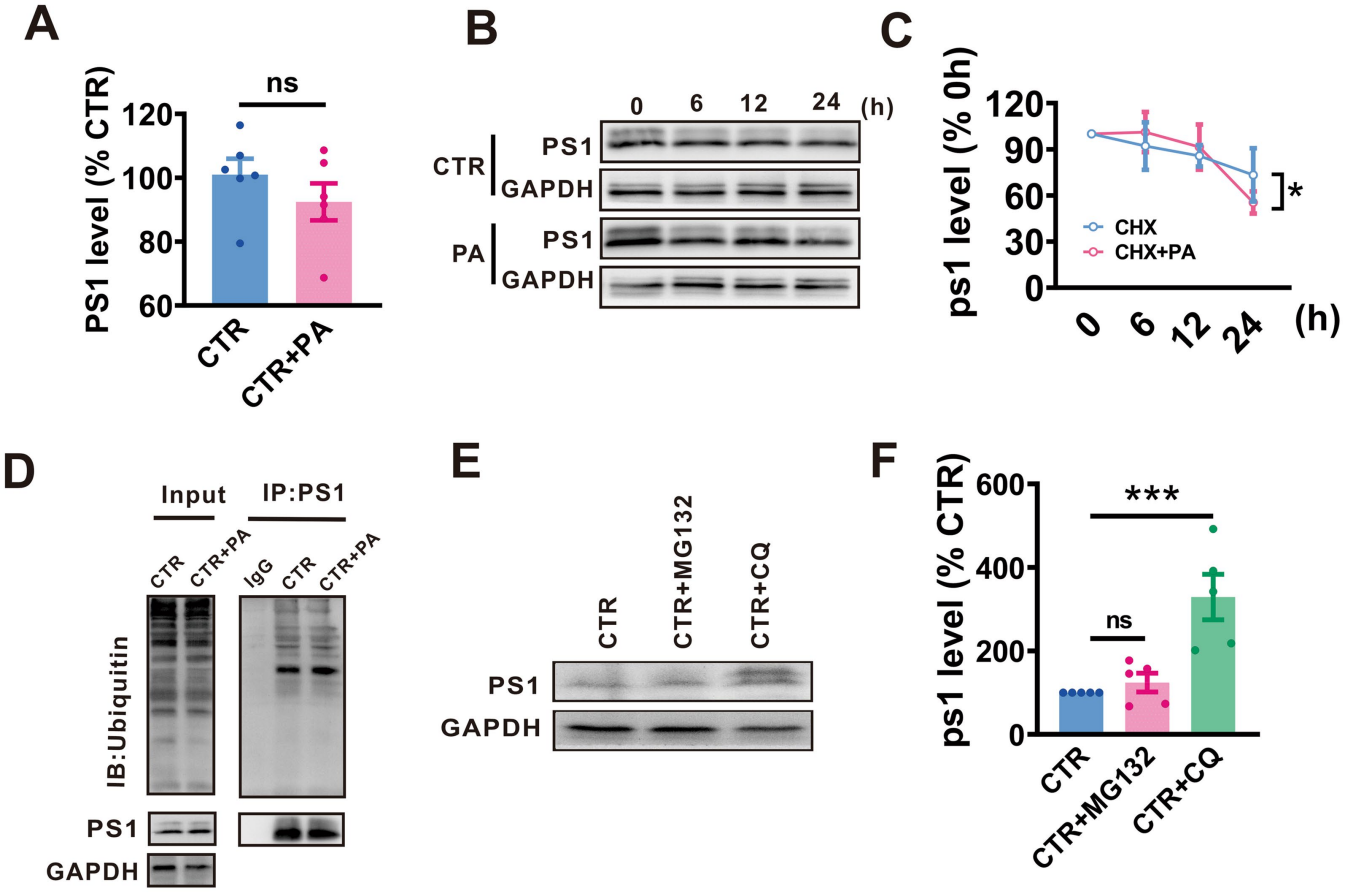
PA promotes autophagy-lysosomal degradation of PS1. **(A)** PS1 mRNA levels assessed by qRT-PCR in N2A^APP^ cells treated with PA (40 μM, 24 h). *n* = 4 in each group. **(B,C)** Effect of PA on PS1 degradation assessed by half-life measurements in N2A^APP^ cells treated with 100 μg/mL cycloheximide (CHX). *n* = 5–6 in each group. **(D)** The total ubiquitination level and the ubiquitination level of PS1 assessed by Western blot and Co-IP in N2A^APP^ cells treated with PA (40 μM for 24 h). *n* = 2 in each group. **(E,F)** The protein level of PS1 assessed by Western blot in N2A^APP^ cells treated with PA (40 μM for 24 h) along with or without 6 h of pretreatment with CQ (50 μM for 24 h) and MG132mg (5 μM for 24 h). *n* = 5 in each group. Data are presented as mean ± standard error, *p < 0.05, **p < 0.01, ***p < 0.001.

Give that protein homeostasis is maintained by a balance between protein synthesis and degradation, we examined whether PA treatment affects either the synthesis or degradation of PS1, thereby influencing its expression levels. To determine the effects of PA on PS1 synthesis, qRT-PCR was utilized to measure PS1 mRNA levels in N2A^APP^ cells following PA treatment. It was observed that PA treatment did not alter PS1 mRNA levels in N2A^APP^ cells (*n* = 6, 92.45% ± 7.630%, *p* = 0.290 vs. 0 μM; Figure 2A).

To examine the impact of PA on the degradation of PS1, a protein synthesis inhibitor, cycloheximide (CHX), was applied to N2A^APP^ cells with or without PA treatment. The results indicated that PA treatment accelerated PS1 degradation compared to controls (*p* = 0.035 vs. CTR; *n* = 5 in each group; Figures 2B,C).

Eukaryotes have two major protein degradation systems, the ubiquitin-proteasome system (UPS) and the autophagolysosome pathway (ALP). To elucidate the potential involvement of the UPS in the observed reduction in APP processing, we employed MG132 to inhibit the proteasome. It was observed no significant change in PS1 ubiquitination levels (*n* = 4 in each group; Figure 2D). To test whether PA influences the degradation of PS1, chloroquine (CQ), an autophagy inhibitor, and MG132, a proteasome inhibitor, were used. CQ treatment resulted in an increase in PS1 expression, whereas MG132 had no effect on PS1 levels (*n* = 5, MG132: 124.10% ± 22.58%, *p* = 0.626 vs. CTR, CTR + CQ: 329.10% ± 54.44%, *p* = 0.001 vs. CTR in each group; Figures 2E,F).

### Autophagy accumulation occurs in AD

**Figure 3:**
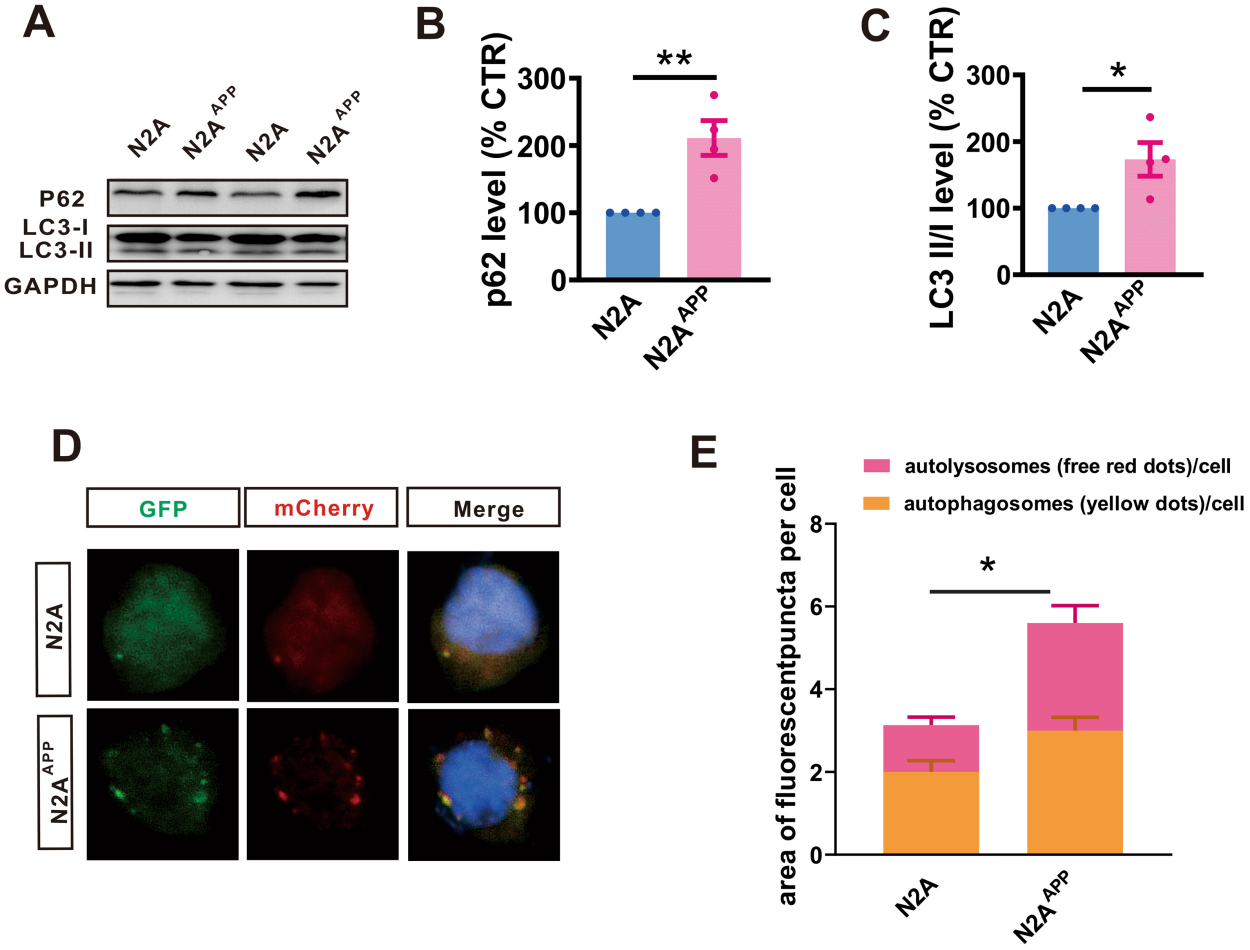
Autophagy accumulation occurs in AD. **(A–C)** The protein levels of P62 **(A,B)**, and LC3 **(A,C)** assessed by Western blot inN2A and N2A^APP^ cells. **(D,E)** The autophagic flux assessed by a fluorescence assay with mCherry-GFP-LC3 in N2A^APP^ cells. *n* = 15–25 in each group. Data are presented as mean ± standard error, *p < 0.05, **p < 0.01, ***p < 0.001.

To assess the effect of PA on autophagy, we examine the autophagy activity in N2A^APP^ cells and compared it with N2A cells. It was found that an increase in autophagic markers, such as P62 (*n* = 4, 221.10% ± 25.90%, *p* = 0.005 vs. N2A; Figures 3A,B) and LC3 II/I (*n* = 4, 173.20 ± 25.21%, *p* = 0.027 vs. N2A; Figures 3A,C) in N2A^APP^ cells, indicating enhanced autophagy accumulation in Alzheimer’s disease. Furthermore, we utilized a fluorescence assay using mCherry-GFP-LC3 to examine the autophagic flux in N2A^APP^ cells. This assay capitalizes on the differential stability of mCherry and GFP fluorescence in acidic environments; mCherry fluorescence persists, whereas GFP fluorescence is quickly quenched. Therefore, an increase in autophagic flux would elevate both yellow (combined mCherry and GFP) and red (only mCherry) puncta, whereas a blockage in autophagosome-lysosome fusion or a rise in lysosomal pH would predominantly increase yellow puncta. Our findings demonstrated a marginal increase in yellow dots (*n* = 16–25, 2.01 ± 0.01, *p* = 0.053 vs. N2A; Figure 3D) and a significant increase in red dots (*n* = 16–25, 2.50 ± 0.43, *p* = 0.006 vs. N2A; Figure 3E), suggesting a blockage in the fusion of autophagosomes with lysosomes or inhibition of lysosomal degradation. These findings indicate an accumulation of autophagy in AD.

### PA enhances the autophagy flux in N2A^APP^ cells

**Figure 4:**
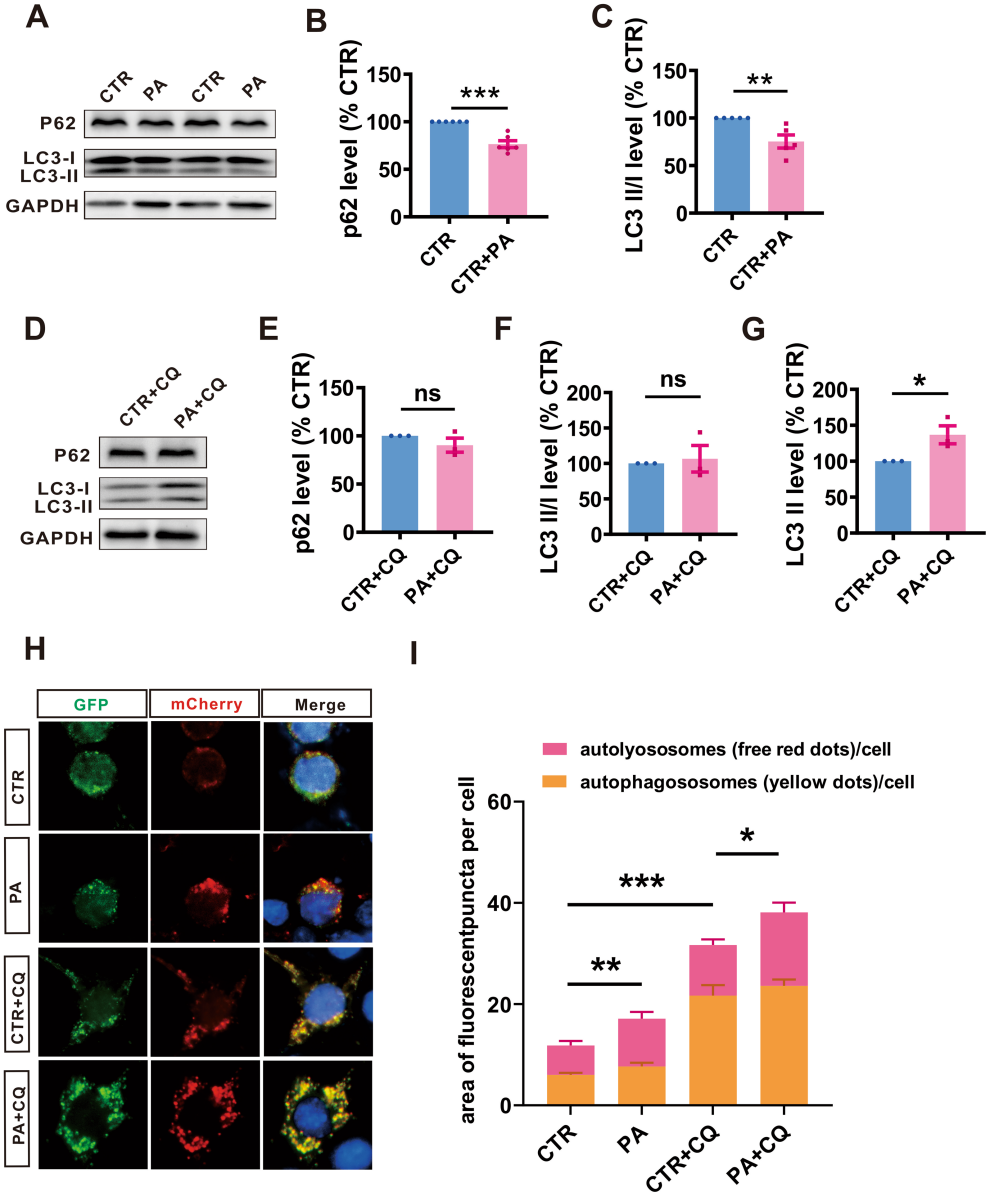
PA enhances the autophagy flux in N2A^APP^ cells. **(A–C)** The protein levels of P62 **(A,B)**, and LC3 **(A,C)** assessed by Western blot in N2A^APP^ cells treated with PA (40 μM for 24 h). *n* = 5–6 in each group. **(D–G)** The protein levels of P62 **(D,E)**, and LC3 **(D,F,G)** assessed by Western blot in N2A^APP^ cells pretreated with PA (40 μM for 6 h) and then co-treated with CQ for 24 h (50 μM). *n* = 4 in each group. **(H,I)** The autophagic flux assessed by a fluorescence assay with mCherry-GFP-LC3 in N2A^APP^ cells pretreated with PA (40 μM for 6 h) and then co-treated with CQ for 24 h (50 μM). *n* = 12–21 in each group. Data are presented as mean ± standard error, *p < 0.05, **p < 0.01, ***p < 0.001.

To explore whether PA affects the autophagolysosome pathway, we treated N2A^APP^ cells with PA and assessed the levels of autophagy-associated proteins P62 and LC3. The results revealed that PA treatment led to a decrease in P62 (*n* = 6, 76.69% ± 3.64%, *p* = 0.001 vs. CTR; Figures 4A,B) levels and LC3 II/I ratios (*n* = 5, 75.19% ± 6.91%, *p* = 0.072 vs. CTR; Figures 4A,C), suggesting that PA may enhance autophagy function in AD model cells.

To further explore PA’s effects on the autophagolysosome pathway, we used CQ to inhibit this pathway and examined the expression of P62 and LC3 in N2A^APP^ cells treated with PA and CQ. The results showed that PA treatment resulted in an increase in LC3 II (*n* = 5, 165.70% ± 28.4%, *p* = 0.049 vs. CTR + CQ; Figures 4D,G) in N2A^APP^ cells. However, PA did not significantly alter P62 expression (*n* = 5, 96.93% ± 17.04%, *p* = 0.861 vs. CTR + CQ; Figures 4D,E) and LC3 II/I ratios (*n* = 5, 163.10% ± 72.71%, *p* = 0.410 vs. CTR + CQ; Figures 4D,F), suggesting that PA may counteract the autophagy inhibition caused by CQ.

Further analysis using a fluorescence assay with mCherry-GFP-LC3 revealed that PA treatment enhanced autophagic flux, as evidenced by an increase in both total (*n* = 11, PA: 17.40 ± 0.58, *p* = 0.001 vs. CTR, *n* = 17, PA + CQ: 39.41 ± 3.15, *p* = 0.008 vs. CTR + CQ in each group; Figures 4H,I) and red puncta (*n* = 12, PA: 9.44 ± 2.67, *p* = 0.178 vs. CTR, *n* = 21, PA + CQ: 14.50 ± 2.01, *p* = 0.030 vs. CTR + CQ in each group; Figures 4H,I), without affecting the yellow puncta (*n* = 12, PA: 7.67 ± 2.67, *p* = 0.533 vs. CTR, *n* = 21, PA + CQ: 23.63 ± 2.00, *p* = 0.331vs. CTR + CQ in each group; Figures 4H,I). In contrast, CQ treatment led to an increase in both total and yellow puncta while slightly reducing red puncta (*n* = 12, CTR + CQ: 10.00 ± 2.23, *p* = 0.064 vs. CTR). Notably, adding PA to CQ-treated cells resulted in an increase in total puncta, emphasizing PA’s potential to reverse CQ’s inhibitory effects on autophagy.

### PA rescues cognitive impairment in Aβ_1–42_-induced mouse model of AD

**Figure 5:**
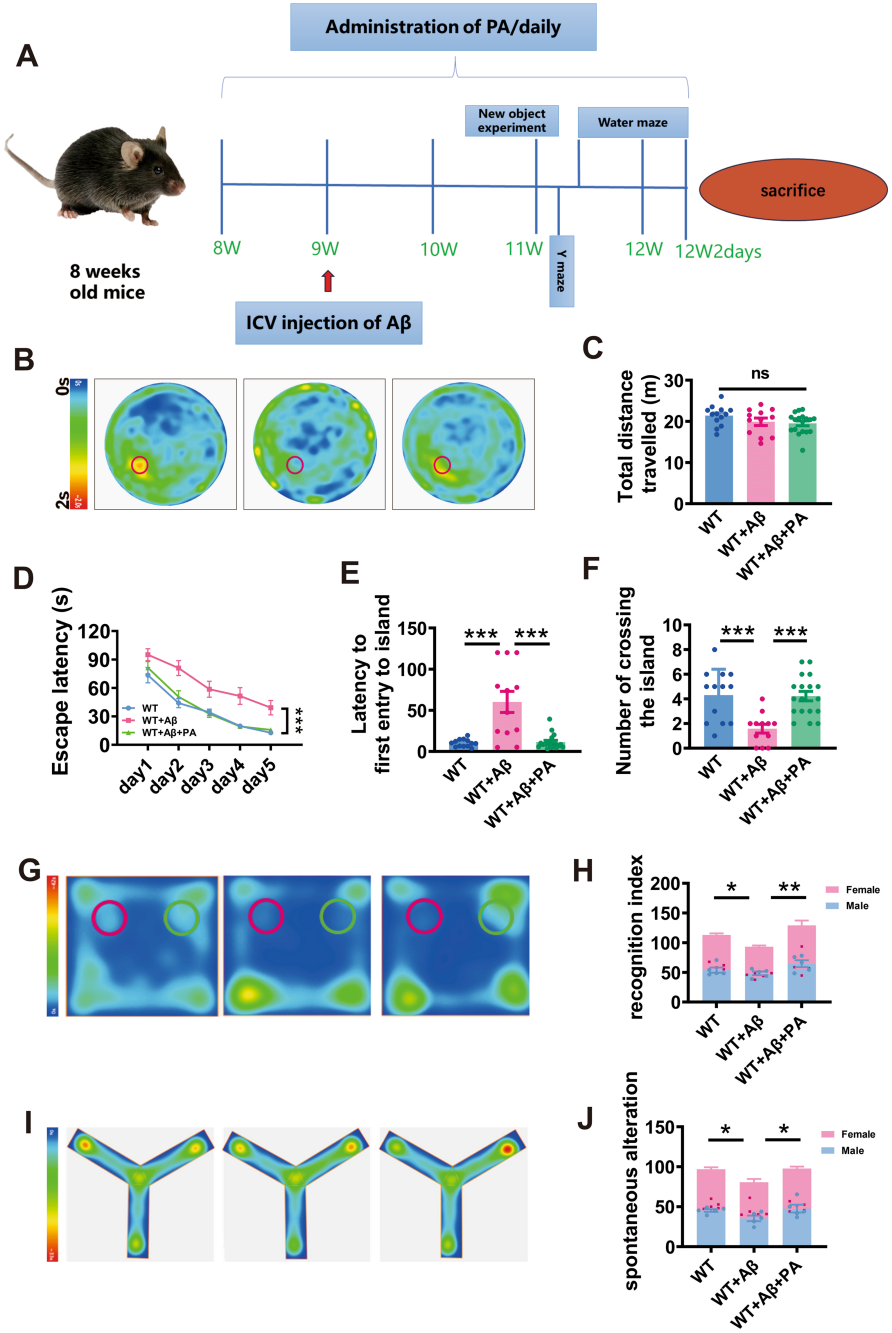
PA attenuates Aβ_1–42_-induced spatial learning and memory deficits in mice. **(A)** Flow chart of animal experiments. **(B–F)** Spatial learning and memory retrieval assessed by Morris water maze test in WT and Aβ-treated mice treated with or without PA treatment. The average speed of travel in the Morris water maze during the adaptation phase **(B)**, the escape latency to the hidden platform a during the spatial learning period **(C)**, first entry into the platform area and the number of entries into the platform zone during the memory retrieval test. *n* = 12–18 in each group. **(G,H)** The cognitive function of mice was evaluated by the novel object recognition test, and the green area was the cognitive coefficient of the novel object recognition area, **(H)** cognitive index; **(I,J)** Spontaneous alter country behavior of mice was assessed by Y-maze, and **(J)** spontaneous alteration. Data representation of average ± SEM, *p < 0.05, **p < 0.01, ***p < 0.001.

Given PA’s role in inhibiting APP processing, we explored its impact on the cognitive functions of AD mouse models. A mouse model was created through microinjection of Aβ_1–42_ into the lateral ventricle of WT mice. We then assessed the therapeutic effects of PA through daily intraperitoneal injections of 10 mg/kg, starting 1 week before and continuing until after the behavioral tests. Two weeks post-Aβ_1–42_ injection, the mice underwent the Morris water maze test to assess their spatial learning and memory (Figure 5A).

During the adaptation period, the average swimming distances were comparable across all groups (WT: 21.39 ± 0.68 m; WT + Aβ: 19.90 ± 0.92 m, *p* = 0.175 vs. WT; WT + Aβ + PA: 19.50 ± 0.59 m, *p* = 0.061 vs. WT + Aβ, *p* = 0.692 vs. WT; *n* = 12–18 in each group; Figures 5B,C), indicating that neither Aβ_1–42_ nor PA treatment affected motor function. During the training phase, mice treated with Aβ_1–42_ showed significant spatial learning deficits, with increased escape latency to locate the hidden platform compared to WT mice. However, PA treatment substantially reduced the escape latency in Aβ_1–42_ treated mice (*p* < 0.001 vs. WT + Aβ; *p* = 0.845 vs. WT; *n* = 12–18 in each group; Figure 5D). In the probe test, which measures memory retrieval, Aβ_1–42_ treated mice demonstrated significantly a delayed latency to first entry into platform-zone (WT: 10.28 ± 1.27 s; WT + Aβ: 60.18 ± 12.82 s, *p* < 0.001 vs. WT; *n* = 12–18 in each group; Figure 5E) and fewer platform-zone crossing (WT: 4.308 ± 0.58; WT + Aβ: 1.58 ± 0.36, *p* = 0.001 vs. WT; *n* = 12–18 in each group; Figure 5F). PA treatment markedly improved these metrics, restoring them to levels similar to the WT group (for first entry into platform-zone: WT + Aβ + PA: 11.41 ± 2.15 s, *p* < 0.001 vs. WT + Aβ, *p* = 0.900 vs. WT, *n* = 12–18 in each group, Figure 4E; for platform-zone crossing: WT + Aβ + PA: 4.22 ± 0.38, *p* = 0.001 vs. WT + Aβ, *p* = 0.890 vs. WT, *n* = 12–18 in each group; Figure 5F).

To further investigate the impact of PA on cognition. Novel object recognition test and Y maze tests were conducted. The findings indicated that the Aβ_1–42_ significantly reduced the recognition index and spontaneous alteration rate (For novel object recognition test: WT: 56.54 ± 2.57; WT + Aβ: 46.63 ± 1.85, *p* = 0.043 vs. WT; *n* = 10 in each group; Figures 5G,H; For Y maze: WT: 48.49 ± 1.70; WT + Aβ: 40.31 ± 3.00, *p* = 0.028 vs. WT; *n* = 10 in each group; Figures 5I,J). However, PA treatment markedly improved these measures, restoring them to levels observed in the WT group (For novel object recognition test: WT + Aβ + PA: 22.80 ± 2.77, *p* < 0.001 vs. WT + Aβ; *n* = 10 in each group; Figures 5I,J; WT + Aβ: 64.58 ± 4.75, *p* < 0.001 vs. WT + Aβ; *n* = 10 in each group; Figures 5G,H; For Y maze: WT + Aβ + PA: 48.88 ± 2.60, *p* = 0.022 vs. WT + Aβ; *n* = 10 in each group; Figures 5I,J). These results suggest that PA effectively counters cognitive decline in AD model mice.

To explore the effect of sex on AD, we counted the cognitive coefficients and spontaneous alterations of female and male mice separately for each group and found no significant difference between the sexes (For novel object recognition test: male: WT: 54.64 ± 4.12; WT + Aβ: 48.38 ± 5.31, *p* = 0.823vs. WT; WT + Aβ + PA: 64.64 ± 12.26, *p* = 0.076 vs. WT + Aβ; female: WT: 59.42 ± 5.13; WT + Aβ: 44.88 ± 4.51, *p* = 0.128 vs. WT; WT + Aβ + PA: 64.52 ± 19.64, *p* = 0.052 vs. WT + Aβ; *n* = 10 in each group; Figure 5H; For Y maze: male: WT: 45.31 ± 9.96; WT + Aβ: 35.34 ± 2.34, *p* = 0.126 vs. WT; WT + Aβ + PA: 47.65 ± 12.31, *p* = 0.053 vs. WT + Aβ; female: WT: 51.66 ± 6.39; WT + Aβ: 45.27 ± 1.55, *p* = 0.460 vs. WT; WT + Aβ + PA: 50.11 ± 4.84, *p* = 0.674 vs. WT + Aβ; *n* = 10 in each group; Figure 5L).

### PA rescues oxidative stress and inflammatory response in Aβ_1–42_-induced mouse model of AD

**Figure 6:**
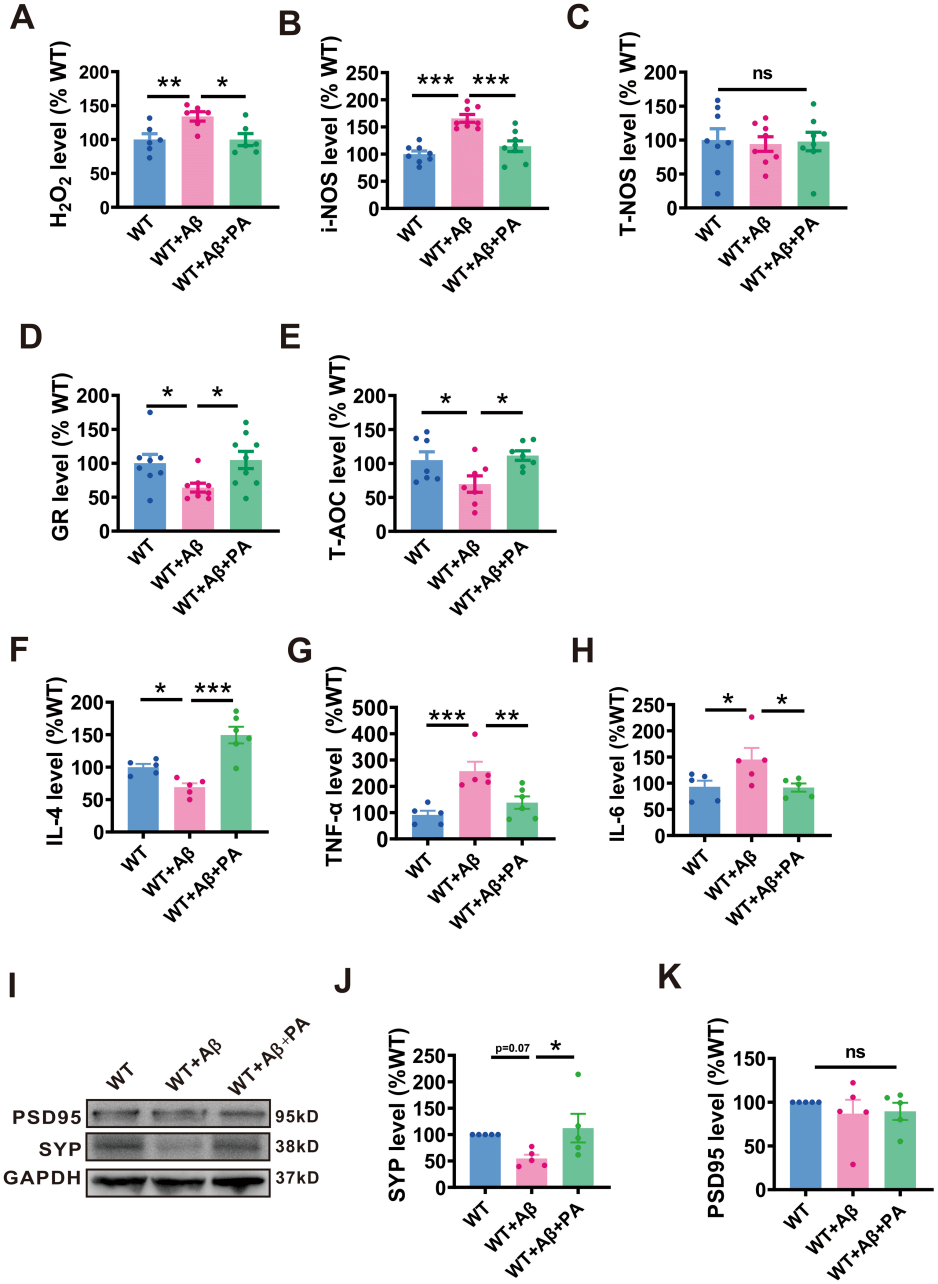
PA reduces oxidative stress in Aβ_1–42_-induced mouse model of AD. **(A–C)** Pro-oxidant H_2_O_2_ **(A)**, i-NOS **(B)**, and T-NOS **(C)** assessed by relevant commercial kits in the brains of Aβ_1–42_ treated mice with or without PA treatment. *n* = 12–18 in each group. **(D,E)** antioxidants GR **(D)** and T- AOC **(E)** assessed by the relevant commercial kits in the brains of Aβ_1–42_ treated mice with or without PA treatment. *n* = 7–13 in each group. PA regulates the levels of inflammatory cytokines in Aβ_1–42_-induced mouse. The levels of inflammatory factors IL-4 **(F)**, TNF-α **(G)** and IL-6 **(H)** in the brain of mice were treated with Aβ, and the changes were evaluated with or without PA by QRT-PCR. Each group is *n* = 5. PA can regulate the level of SYP protein in Aβ_1–42_-induced mouse **(I)**. The protein level of SYP **(J)**, and PSD95 **(K)** assessed by Western blot in Aβ_1–42_-induced mouse *n* = 5 each group. Data are presented as mean ± standard error, *p < 0.05, **p < 0.01, ***p < 0.001.

Oxidative stress is a significant pathological consequence of Aβ pathology and plays a crucial role in the pathogenesis of AD. We therefore investigated the potential role of PA in oxidative stress within an Aβ_1–42_-induced AD mouse model. Our results indicated significant elevations in pro-oxidants levels including hydrogen peroxide (H_2_O_2_) (134.00% ± 6.91%, *p* < 0.01 vs. WT; *n* = 5–8 in each group; Figure 6A) and inducible nitric oxide synthase (i-NOS) (165.8% ± 7.08%, *p* < 0.001 vs. WT; *n* = 8 in each group; Figure 6B) in mice treated with Aβ_1–42_. In contrast, antioxidants levels, such as glutathione reductase (GR) (64.13% ± 6.53%, *p* = 0.038 vs. WT; *n* = 11–13 in each group; Figure 6D) and total antioxidant capacity (T-AOC) (69.68% ± 12.11%, *p* < 0.033 vs. WT; *n* = 7–8 in each group; Figure 6E) were significantly decreased in these mice. Notably, PA treatment effectively normalized both the increased pro-oxidants and the reduced antioxidants levels (for H_2_O_2_: 99.77% ± 8.94%, p < 0.001 vs. WT + Aβ, *p* = 0.985vs. WT, *n* = 5–8 in each group, Figure 6A; for i-NOS: 114.6% ± 9.08%, *p* = 0.001vs. WT + Aβ, *p* = 0.197 vs. WT, *n* = 8 in each group; Figure 6B; for GR: 104.8% ± 12.65%, *p* = 0.017 vs. WT + Aβ, *p* = 0.771 vs. WT, *n* = 11–13 in each group; Figure 6D; for T-AOC: 111.5% ± 7.01%, *p* < 0.013 vs. WT + Aβ, *p* = 0.661 vs. WT, *n* = 7–8 in each group; Figure 6E). However, neither Aβ_1–42_ nor PA treatment had an impact on the levels of total nitric oxide synthase (T-NOS) (WT + Aβ: 94.16% ± 10.77%, *p* = 0.770 vs. WT; WT + Aβ + PA: 97.73% ± 13.63%, *p* = 0.858 vs. WT + Aβ, *p* = 0.909vs. WT; *n* = 8 in each group; Figure 6C). Thus, these results affirm the anti-oxidative properties of PA in reducing oxidative stress in AD model mice.

We also explored the anti-inflammatory response of PA in a mouse model of Aβ_1–42_ induced AD. Using qRT-PCR analysis, we observed that interleukin 4 (IL-4), an anti-inflammatory cytokine, was significantly reduced to 69.14% ± 6.01% (*p* = 0.04 vs. WT; *n* = 5 per group; Figure 6F) compared to the AD group. Pro-inflammatory cytokines such as tumor necrosis factor-alpha (TNF-α) and interleukin 6 (IL-6) were notably increased in the AD group, with TNF-α reaching 257.50% ± 35.73% (*p* < 0.001 vs. WT; *n* = 5 per group; Figure 6G) and IL-6 at 145.20% ± 22.01% (*p* = 0.032 vs. WT; *n* = 5 per group; Figure 6H). Notably, PA treatment significantly elevated IL-4 levels to 149.40% ± 12.85% (*p* < 0.001 vs. WT + Aβ + PA; *n* = 5 per group; Figure 6F) and decreased the levels of pro-inflammatory cytokines, with TNF-α reduced to 137.80% ± 23.50% (*p* = 0.006 vs. WT + Aβ + PA; *n* = 5 per group; Figure 6G) and IL-6 to 91.80% ± 7.88% (*p* = 0.028 vs. WT + Aβ + PA; *n* = 5 per group; Figure 6H), highlighting its potential therapeutic benefits in reducing inflammation in AD.

To assess whether PA could alleviate synaptic dysfunction in AD, we examined the levels of synaptic-related proteins, including postsynaptic density protein 95 (PSD95) and synaptic vesicle protein (SYP). Our findings showed that while there was no significant change in PSD95 levels among the groups (*n* = 6, WT + Aβ: 86.98% ± 15.78%, *p* = 0.408 vs. WT: 89.5% ± 9.87%, *p* = 0.869 vs. WT + Aβ + PA; Figures 6I,J), SYP levels were notably reduced in Aβ-treated mice. However, PA treatment was able to rescue SYP levels (*n* = 6, WT + Aβ: 54.7% ± 7.10%, *p* = 0.071 vs. WT: 112.10% ± 27.07%, *p* = 0.027vs. WT + Aβ + PA; Figures 6I,K), suggesting that PA has the potential to improve synaptic dysfunction in AD.

### PA decreased the expression of phosphorylated Tau

**Figure 7:**
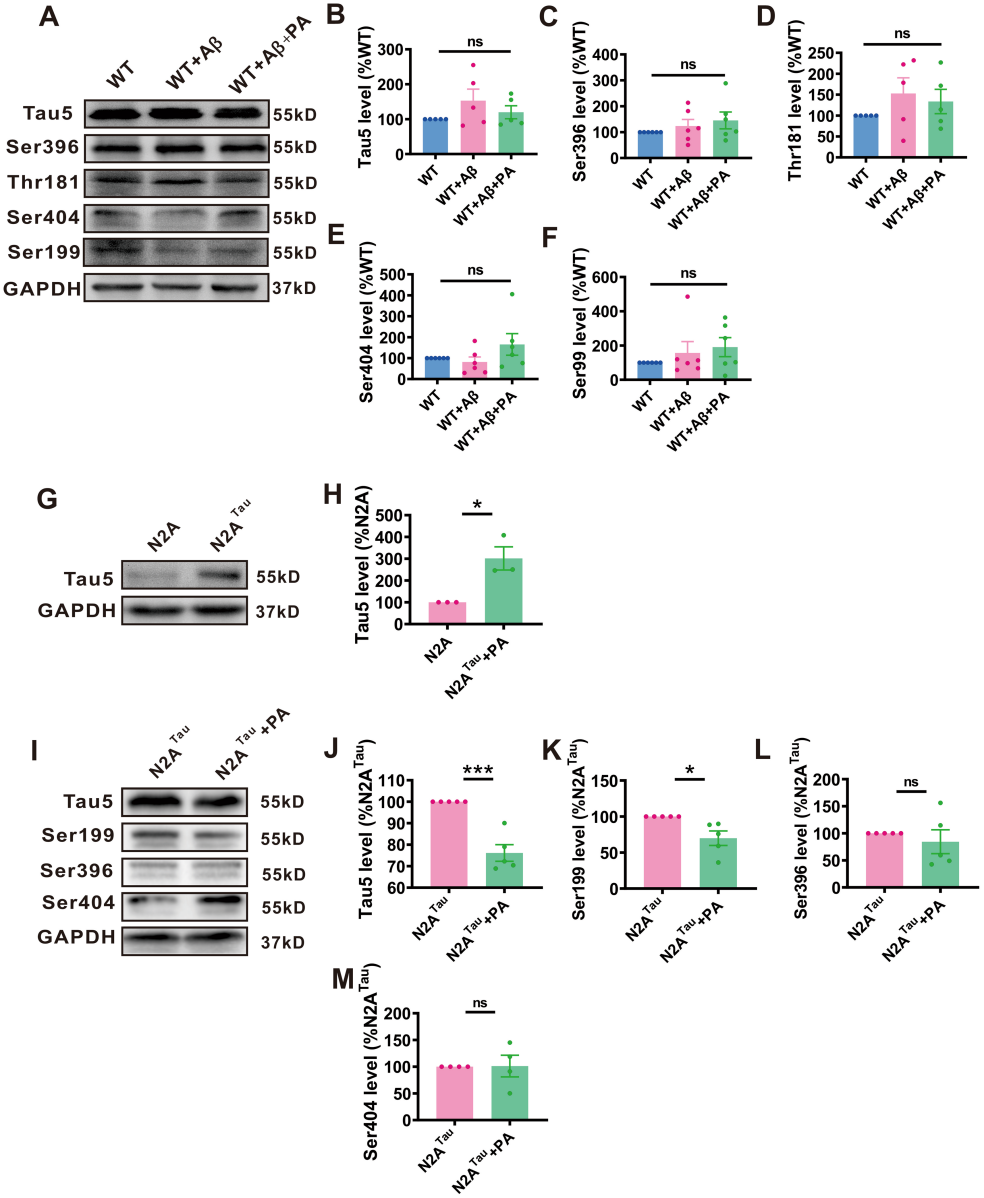
A regulated protein levels of total tau and phosphotau in N2A^Tau^cell lines but not in Aβ_1–42_-induced mouse. The protein level of Tau5 **(B)**, Ser396 **(C)**, Thr181 **(D)**, Ser404 **(E)** and Ser199 **(F)** assessed by Western blot in Aβ_1–42_-induced mouse *n* = 5 each group. The protein level of Tau5 **(H)** assessed by Western blot in N2A^Tau^cell. *n* = 3 in each group. The protein level of Tau5 **(J)**, Ser199 **(K)**, Ser396 **(L)**, and Ser404 **(M)** assessed by Western blot in N2A^Tau^cell. *n* = 5–6 in each group. Data representation of average ± SEM, Aβ_1–42_ Levels of total tau and phosphotau in mice induced by Aβ_1–42_ after PA treatment **(A)**; Levels of total tauin N2A^Tau^cells **(G)**; Levels of total tau and phosphotau in N2A^Tau^cells after PA treatment **(I)**. *p < 0.05, **p < 0.01, ***p < 0.001.

We explore whether PA affects both total Tau and phosphorylated Tau expression. In mice induced with Aβ, no significant changes were observed in levels of total Tau (*n* = 6, WT + Aβ: 152.80% ± 33.64%, *p* = 0.116 vs. WT: 119.80% ± 18.64%, *p* = 0.309 vs. WT + Aβ + PA; Figures 7A,B) or phosphorylated Tau at various sites (Ser396: *n* = 6, WT + Aβ: 123.50% ± 25.54%, *p* = 0.497 vs. WT, 145.30% ± 32.49%, *p* = 0.528 vs. WT + Aβ + PA; Figures 7A,C, Thr181: WT + Aβ: 152.70% ± 37.63%, *p* = 0.199 vs. WT, 136.60% ± 29.08%, *p* = 0.631 vs. WT + Aβ + PA; Figures 7A,D, Ser404: WT + Aβ: 81.54% ± 24.03%, *p* = 0.697 vs. WT, 165.60% ± 51.51%, *p* = 0.091 vs. WT + Aβ + PA; Figures 7A,E, Ser199: WT + Aβ: 156.70% ± 66.57%, *p* = 0.436 vs. WT, 190.60% ± 55.74%, *p* = 0.640 vs. WT + Aβ + PA; Figures 7A,F). This may be due to the inability of endogenous Tau in mice to form neurofibrillary tangles.

To further explore PA’s effect on human Tau, we utilized stabilized N2A cells transfected with human Tau, which successfully and stably overexpressed tau5 (*n* = 3, N2A^Tau^: 301.50% ± 53.18%, *p* = 0.019 vs. N2A; Figures 7G,H). The results demonstrated that PA treatment significantly reduced the expression of Tau5 (*n* = 5, N2A^Tau^: 90.63% ± 10.55%, *p* = 0.002 vs. N2Atau + PA; Figures 7I,J) and phosphorylated Tau at Ser199 (*n* = 5, N2A^TTau^: 69.89% ± 0.07%, *p* = 0.017 vs. N2A^Tau^ + PA; Figures 7I,K). However, no significant changes were noted in the phosphorylation levels at Ser396 (*n* = 5, N2A^Tau^: 84.41% ± 21.99%, *p* = 0.499 vs. N2A^Tau^ + PA; Figures 7I,L) and Ser404 (*n* = 5, N2A^Tau^: 101.20% ± 20.29%, *p* = 0.954 vs. N2A^Tau^ + PA; Figures 7I,M). These data suggest that PA has a mitigating effect on the levels of both total Tau and phosphorylated Tau at various phosphorylation sites.

## Discussion

In the current study, we have demonstrated that PA treatment attenuates the APP processing by promoting the PS1 degradation via the autophagy-lysosome pathway. Furthermore, our results indicate that PA facilitates an increase in autophagic activity by enhancing autophagy flux. Most notably, our data reveal that PA treatment significantly ameliorates cognitive deficits in a mouse model of AD induced by Aβ_1–42_, likely through the alleviation of oxidative stress. Collectively, these findings substantiate the neuroprotective properties of PA in AD, suggesting that PA could be a promising therapeutic candidate for treating AD.

The deposition of Aβ, which originates from the sequential cleavage of APP by β-secretase and γ-secretase, is a primary event in the progression of AD (Jiang et al., 2024). γ-Secretase, comprising PS1 and PS2, nicastrin, presenilin enhancer 2 (PEN-2), and anterior pharynx defective-1 (APH-1) (De Strooper and Annaert, 2010). Substantial research has established that PS1 serves as the catalytic core of the γ-secretase complex, and its functionality is crucial as deficiencies in presenilins are known to inhibit the cleavage of APP and consequently affect Aβ production. In line with these observations, our findings demonstrate that PA reduces Aβ production by lowering PS1 expression (Kelleher 3rd and Shen, 2017).

Previous research has demonstrated that PS1 degradation can proceed via the ubiquitin-proteasome system and autophagy-lysosome pathway (Pasternak et al., 2003; Thinakaran and Koo, 2008). The ubiquitin-proteasome system, central to the degradation of misfolded or aberrantly modified proteins, involves interactions with E3 ubiquitin ligases such as SEL-10 and tumor necrosis factor receptor-associated factor 6 (TRAF6) (Pasternak et al., 2003), which modulate PS1 stability. In our experiments with N2A^APP^ cells treated with both PA and MG132, a proteasome inhibitor, no changes in PS1 ubiquitination levels were observed, suggesting that the reduction in PS1 expression with PA treatment does not involve the ubiquitin-proteasome system. Furthermore, PS1 possesses a ubiquitin-binding CUE domain that specifically enhances its interaction with K63-linked polyubiquitin chains, thus facilitating its regulation via these degradation pathways. Proteins that are ubiquitinated with K63 chains, including PS1, serve as substrates for autophagy receptors such as p62/SQSTM1, which facilitate their transport to the autophagosome (Lee et al., 2010). Consistent with these mechanisms, we observed that inhibition of autophagy by CQ led to an increased expression of PS1, further highlighting the significance of autophagy in regulating PS1 expression.

Autophagy, a crucial cellular degradation process, plays a pivotal role in the accumulation of Aβ and tau proteins in AD (Zhao et al., 2025). Dysfunctional autophagy disrupts the efficient clearance of Aβ peptides, facilitating the formation of amyloid plaques (Wani et al., 2021). Moreover, autophagic vacuoles have been identified as sites of Aβ generation, potentially contributing to its extracellular deposition (Mizushima, 2005). Concurrently, lysosomal anomalies impede the degradation of hyperphosphorylated tau and Aβ, culminating in the formation of senile plaques and neurofibrillary tangles that exacerbate neuronal toxicity (Kim et al., 2025). Emerging evidence underscores the pivotal function of autophagy in both the formation and clearance of these protein aggregates. Activation of autophagic pathways has been shown to exert protective effects in animal models of tauopathy, including mouse and fly systems (Berger et al., 2006; Caccamo et al., 2010). Furthermore, enhancing autophagic activity is posited to ameliorate tau aggregate formation and reduce Aβ accumulation, thereby promoting cellular survival and potentially mitigating the progression of AD (Wang et al., 2023; Wani et al., 2021).

Some evidence shows that autophagosomes and autolysosomes were accumulated in patient brains (Jung et al., 2010; Kim et al., 2011). In our AD model cells (N2A^APP^), elevated levels of LC3 and P62 were observed. While PA has not been directly shown to enhance autophagy, recent studies indicate that it inhibits mTOR signaling, which negatively regulates autophagy initiation (Lin et al., 2016; Yang et al., 2019). This aligns with our findings that PA treatment resulted in decreased P62 expression in N2A^APP^ cells, suggesting a potential promotion of autophagy. Moreover, CQ, which is known to inhibit autophagy by disrupting autophagosome-lysosome fusion and elevating lysosomal pH, was found to have its effects mitigated by PA. Co-treatment with CQ and PA enhanced autophagic flux as reflected by increase LC3 II levels. PA may potentially enhance lysosomal acidification or promote lysosomal enzyme activity, or it may directly facilitate the autophagosome-lysosome fusion process. Additional investigations are necessary to delineate the precise biochemical interactions between PA and CQ.

The brain, as an organ with high metabolic demands, relies on the continuation of redox reactions to generate the necessary energy for its diverse biochemical functions. This requirement renders the brain particularly susceptible to oxidative stress (Manoharan et al., 2016). Reactive oxygen species, at physiological levels, are crucial for the maintenance of cellular homeostasis. However, an elevation in ROS levels, such as hydrogen peroxide, represents a significant event in the pathogenesis of AD (Roy et al., 2023). Research in both *in vitro* and *in vivo* models has demonstrated that Aβ peptide and tau phosphorylation independently contribute to the elevation of ROS, thereby precipitating oxidative stress (Cheignon et al., 2018). Aβ oligomers disrupt the functionality of N-methyl-D-aspartate (NMDA) receptors, leading to an overproduction of extracellular ROS and an aberrant influx of calcium into neurons (Sutherland et al., 2013). Further investigations reveal that oxidative stress significantly enhances the activity of BACE1 through the activation of the c-Jun N-terminal kinase (JNK)/Activator Protein 1 (AP1) signaling pathway (Tamagno et al., 2012). The consequent oxidative environment not only supports tau phosphorylation but also fosters the development of neurofibrillary pathologies. Within the context of tauopathies, oxidative stress is implicated in inducing JNK-mediated apoptotic pathways and disturbances in the neuronal cell cycle (Dias-Santagata et al., 2007). Therapeutic strategies aimed at mitigating oxidative stress have shown potential in reducing both Aβ aggregation and tau-associated neuropathologies (Bai et al., 2022).

In AD, excessive release of ROS occurs due to positive ions at the hydrophilic N-terminus of Aβ, leading to oxidative stress (Cioffi et al., 2019; Mattson, 2004; Wan et al., 2011; Zhu et al., 2006). Although PA has not been directly shown to possess antioxidant properties, its structural analog, Parishin C, has demonstrated efficacy in reducing oxidative stress in cerebral ischemia (Wang et al., 2021; Zhan et al., 2016; Zhu et al., 2023). Given PA’s regulatory effects on inflammation via M2 (Xu et al., 2024) macrophage polarization and its potential antioxidative properties, it is plausible that PA could ameliorate cognitive dysfunction in Aβ_1–42_-induced mice through promoting autophagy and inhibiting oxidative stress, positioning PA as a potential therapeutic agent for AD.

Aβ oligomers are widely recognized as the principal neurotoxic entities in AD, implicated in inducing synaptic dysfunction and neurodegeneration. Utilizing the Aβ injection model enables the focused investigation of the acute neurotoxic effects of Aβ oligomers. These oligomers are challenging to isolate in transgenic models due to the intricate and prolonged nature of the pathology. In contrast, transgenic AD models exhibit a progressive development of pathology over several months, necessitating extended periods of study. The Aβ injection model offers a more rapid and controllable environment, facilitating the examination of potential therapeutic agents’ effects over shorter durations. This methodology is particularly advantageous for the preliminary screening of neuroprotective compounds like PA prior to their evaluation in more elaborate and time-intensive models. While the Aβ model is valuable for exploring acute Aβ toxicity, it does have limitations in fully replicating the comprehensive pathology of AD, including aspects like tau aggregation and neuroinflammation. The Aβ acute model may also be the reason why we did not see a significant sex difference in our study, because the injection time was 8 weeks, when the sex difference itself was relatively small, unlike in transgenic mice. For example, APP/PS1 double-transfer mice, many literatures show that behavioral cognitive impairment and learning and memory impairment have been detected at 10 or 12 months (Li et al., 2016; Wang et al., 2003), or there is a significant gender difference in the transcriptome profile is already 8 months of age (Papazoglou et al., 2024). Subsequent research endeavors will integrate transgenic AD models to corroborate our findings within a more chronic setting of the disease, thereby augmenting the translational relevance of our research.

## Conclusion

This study indicates that PA treatment alleviates APP processing by promoting the PS1 degradation via the autophagy-lysosome pathway. Additionally, PA treatment significantly ameliorates cognitive deficits in an Aβ_1–42_ induced AD mouse model by mitigating oxidative stress and inflammatory response. These findings support the neuroprotective effects of PA in AD, indicating that PA could be a valuable therapeutic candidate for the treatment of AD.

## Data Availability

The original contributions presented in the study are included in the article/supplementary material, further inquiries can be directed to the corresponding author.
